# Emerging Translational Opportunities in Comparative Oncology With Companion Canine Cancers: Radiation Oncology

**DOI:** 10.3389/fonc.2019.01291

**Published:** 2019-11-22

**Authors:** Michael W. Nolan, Michael S. Kent, Mary-Keara Boss

**Affiliations:** ^1^Department of Clinical Sciences, North Carolina State University, Raleigh, NC, United States; ^2^Comparative Medicine Institute, North Carolina State University, Raleigh, NC, United States; ^3^Duke Cancer Institute, Duke University, Durham, NC, United States; ^4^Department of Surgical and Radiological Sciences, School of Veterinary Medicine, University of California, Davis, Davis, CA, United States; ^5^Department of Environmental and Radiological Health Sciences, Flint Animal Cancer Center, Colorado State University, Fort Collins, CO, United States

**Keywords:** radiation oncology, radiobiology, canine comparative radiation oncology, medical physics, animal models of cancer, imaging, theranostics, translational research

## Abstract

It is estimated that more than 6 million pet dogs are diagnosed with cancer annually in the USA. Both primary care and specialist veterinarians are frequently called upon to provide clinical care that improves the quality and/or quantity of life for affected animals. Because these cancers develop spontaneously in animals that often share the same environment as their owners, have intact immune systems and are of similar size to humans, and because the diagnostic tests and treatments for these cancers are similar to those used for management of human cancers, canine cancer provides an opportunity for research that simultaneously helps improve both canine and human health care. This is especially true in the field of radiation oncology, for which there is a rich and continually evolving history of learning from the careful study of pet dogs undergoing various forms of radiotherapy. The purpose of this review article is to inform readers of the potential utility and limitations of using dogs in that manner; the peer-reviewed literature will be critically reviewed, and current research efforts will be discussed. The article concludes with a look toward promising future directions and applications of this pet dog “model.”

## Introduction

Radiotherapy (RT) is most frequently applied to pet animals with naturally-occurring cancer as a means for improving animal health. However, there is also a long and rich history of studying radiation responses in the tumors and normal tissues of dogs, with a goal of informing therapeutic development in a manner that directly benefits both canine and human cancer patients. Such research forms the basis for the field of canine comparative radiation oncology and radiobiology. The purpose of this review article is to inform readers of the potential utility and limitations of using dogs in that manner; the peer-reviewed literature will be critically reviewed, and current research efforts will be discussed. The article will conclude with a look toward promising future directions and applications of this pet dog “model”; but we will begin with a brief overview of current practices in veterinary radiation oncology, which will enable readers to gain an appreciation for how the field of canine comparative radiation oncology has developed, and how canine cancer studies can be efficiently, effectively and ethically conducted.

The American Veterinary Medical Association estimated that 38% of households in the Unites States owned a pet dog in 2018, and the National Cancer Institute's Comparative Oncology Program reports that nationwide ~6 million new canine cancer diagnoses are made annually. RT is an important component of cancer care for dogs; external beam RT is the most common form of treatment. But despite the high rate of canine cancers, and the efficacy of RT, it is actually quite uncommon for dogs to be treated as such; this is largely attributable to limited accessibility. Financial cost is another important barrier to pet owners who might otherwise want to pursue advanced cancer treatments. The economics of veterinary RT are not well-documented. The cost of care varies with geographic location and type of center (university run academic veterinary teaching hospitals vs. private practice specialty clinics), but anecdotally, a course of palliative-intent RT in the US currently costs between ~$1,000 and $3,500, and the cost for a definitive-intent course of therapy is often between $5,000 and $12,000. This is challenging because fewer than 10% of households carry pet health insurance, and so for most families, veterinary care represents an out-of-pocket expense. Another important factor that limits accessibility is the distance that must be traveled to pursue care; a 2010 report identified 66 veterinary-specific external beam RT centers in the United States (1). While that number is low relative to the number of canine cancer cases, geographic access is improving; indeed, there were only 42 US-based veterinary RT centers in 2001 (2).

Dogs develop a wide range of neoplasms that are treated with RT. Some of the most common indications with translational relevance to humans include soft tissue sarcoma, extremity osteosarcoma, glioma, and genitourinary carcinomas (both muscle-invasive urothelial carcinomas of the urinary bladder, and prostatic carcinomas). Many of these canine cancers share striking similarities with the equivalent human diseases, not only clinically and histologically, but also at a molecular and genomic level. For example, the most commonly altered gene in both canine and human osteosarcoma is TP53; the genomic imbalance in the two species is similar, and expression of several genes (including PTEN and RUNX2) are correlated with ploidy (3–5). Likewise, in addition to certain similarities between human and canine glioma, the process of PDGF-induced gliomagenesis seems to be well-conserved across mammalian species (6, 7).

A majority of canine radiotherapy patients are treated using conventional C-arm linear accelerators. Due to the need for general anesthesia, full-course definitive-intent, treatment protocols tend to be hypofractionated relative to protocols in common use for humans; often a total of 16–20 daily (Monday through Friday) fractions are administered, with fractional doses used for dogs typically ranging from 2.5 to 4 Gy. As with physician-based oncology, there has also been a tremendous shift in recent years toward much higher doses per fraction, with a rapid increase in access to, and application of, stereotactic radiosurgery (SRS) and stereotactic body RT (SBRT) for various malignancies (1).

## Historical Uses of Dogs in Translational Radiation Research

### Normal Tissue Toxicity

In an online query of the US National Library of Medicine's PubMed database for the terms “canine” and “radiation,” the earliest return was a 1922 article that was written by Stafford Warren and George Whipple; it was published in the Journal of Experimental Medicine, and described small intestinal radiosensitivity in dogs (8). This early use of dogs for radiobiology research may have been driven at least in part by the ease of housing and handling dogs, as well as access to hospital-grade irradiation equipment (X- and gamma-ray) for which it would have been straightforward to perform partial or total body irradiations. The similar anatomy of dogs and humans would also have been favorable, thus allowing experiments to be performed with similar radiation field sizes, and targets, as compared with other research species of the times, including various large animal agricultural species (e.g., swine, cattle) and small fish (9–11). Fortuitously, it was later learned that DNA repair mechanisms are highly conserved between mammalian species, and that there is high homology between key DNA damage response genes in humans and dogs (12–14).

Dogs have been used to model radiation injury in a wide range of normal tissues. And indeed, just as veterinarians have borrowed from physicians to inform the practice of veterinary radiation oncology, lessons learned from canine radiobiology research have also had important influences to optimize human cancer care. Through the 1980's and 1990's significant efforts at both Colorado State University and the National Cancer Institute (NCI) were directed toward defining the tolerance of canine tissues to intra-operative RT (IORT); similar studies were performed with multi-fraction irradiation protocols to estimate alpha-to-beta ratios for various normal tissues. These studies evaluated both conventional pathologic endpoints, and a wide range of clinically-relevant functional endpoints. For example, in a canine study, the volume of lung irradiated was found to be a critical limiting factor in thoracic RT (15). The assumption had already been made that a dose-volume relationship existed for lung tissue (16); the TD_50/5_ (the radiation dose that causes toxicity in 50% of patients, 5 years after irradiation) was estimated to be 65 Gy when 33% of the total lung volume was irradiated. However, there were no cases of severe symptomatic pneumonitis in dogs for whom 33% of lung received up to 72 Gy; this suggests that when patients have otherwise healthy lungs (with normal compensatory function) the dose tolerance limits for lung may actually be higher than those originally proposed by Emami et al. (16).

It is beyond the scope of this manuscript to review all normal tissue injury studies for which dogs were utilized; however, another noteworthy endeavor was the use dogs to investigate tolerance of peripheral nerves to IORT. In the 1990's, investigators at NCI demonstrated a progressive sciatic neuropathy in American Foxhounds: with 3.5 years of follow-up, doses of up to 20 Gy were not found to cause clinically significant neuropathies, but 5 years after irradiation, doses of 15 Gy or more of IORT were injurious (17, 18). Similar findings were reported in beagles, whose nerve function was evaluated via histology, neurologic examination, and electrophysiology (19). These experiments confirmed that nerves are dose-limiting for IORT, and that the neurovasculature plays a critical role in nerve injury. These studies also yielded insight regarding combinatorial therapies; while combining IORT with external beam RT resulted in a similar incidence and latency of neuropathy vs. IORT alone, the combination of IORT with hyperthermia led to increased incidence and decreased time to onset of nerve damage. More recently, hounds were used to demonstrate that both altered function of the internal pudendal artery, and pudendal nerve, accompany erectile dysfunction that follows treatment of prostate cancer with SBRT (20). That study identified potential strategies for mitigating radiation-induced sexual dysfunction in men. It also provided data to support the clinical observation that high-dose SBRT may lead to severe colorectal injury, for which latency can be increased, and incidence reduced via either reduction of the radiation dose, or increasing the duration of the interfraction interval (21, 22).

While some laboratory groups have studied both purpose-bred and tumor-bearing pet dogs, the latter have largely been used to study radiation effects on tumors, rather than normal tissue. Rainer Storb's team at the Fred Hutchinson Cancer Research Center has taken this approach for many years, demonstrating dose, fractionation and dose rate effects of total body irradiation in research colony animals, and providing proof-of-principle for clinical applications in the management of lymphomatous diseases in pet dogs (23–36). With rising clinical interest in bone marrow transplantation (including total body irradiation for marrow ablation) in pet dogs with high grade (non-Hodgkin's) multicentric lymphomas, opportunities exist to test novel radiomitigators (37). Certainly, infrastructure exists to enable canine clinical trials to study radiation-modifying drugs and devices. A classic example is a 1986 paper describing the role of canine comparative radiation oncology research in early development of the FDA-approved radioprotector amifostine (WR-2721); a bi-institutional canine clinical trial was performed, in which 73 pet dogs with spontaneously occurring soft tissue sarcomas were randomized (over a period of 3 years) to two dose response assays to receive irradiation alone, or with the radioprotector WR-2721 (38). With the dose and schedule used (40 mg/kg given intravenously, 15 min before each of 10 radiation fractions), WR-2721 provided no protection against acute skin reactions, little to no protection against late complications, and there was a suggestion that the drug protocol provided protection of the tumors at the low end of the radiation dose range. A contemporary example of how a similar study design can be deployed is provided by ongoing research by two authors of this review; both Nolan and Boss are involved in an NIH-sponsored bi-institutional canine clinical trial (5R01CA232148-02) which is currently enrolling 104 dogs with spontaneously occurring soft tissue sarcomas (over a 5 year period) to determine the safety of, and radiation enhancement provided by ultrasound-guided oxygen release from microbubbles. Conduct of these large-scale studies has historically been overseen and facilitated by clinical study coordinators and study teams at the local institutions. Today, large-scale multi-center trials can be coordinated by the Comparative Oncology Program (COP), which is a core resource of the Center for Cancer Research at the National Cancer Institute. The COP centrally manages a network of 20 academic comparative oncology centers; these centers comprise the Comparative Oncology Trials Consortium (COTC). Similarly, and with support from the V-Foundation, a second trials network is currently being planned. As envisioned, that group, called the Canine Oncology Research Consortium (CORC), will include multiple academic partners, with each partner defined as an academic veterinary center paired with an NCI-designated Comprehensive Cancer Center. Both the COP/COTC and CORC exemplify the expanded support that is now available for efficient conduct of canine comparative oncology clinical trials.

Another contemporary example of how normal tissue toxicity data can be directly gathered from pet dogs is provided by an author of this review; while not yet published, Nolan et al., have presented an abstract which describes how pain can be measured and modeled in dogs in pet dogs that develop acute radiodermatitis while undergoing post-operative RT for incompletely excised extremity soft tissue sarcomas (39). Their work not only indicated that mechanical quantitative sensory testing can be used as a reliable tool for preclinical evaluation of novel analgesic strategies, but also provides evidence that localized RT-induced pain is accompanied by widespread somatosensory sensitization. That set of experiments provides a template for how pet dog studies might fit into the traditional paradigm of therapeutic development: (1) hypothesis generating observations can first be made in people or dogs undergoing cancer therapy; (2) mechanistic studies can then be performed in more conventional preclinical models; (3) novel therapies that arise from the preclinical work can then be efficiently testing in dogs, to provide proof-of-concept for safety and clinical efficacy, before advancing to early phase human clinical trials.

### Tumor Microenvironment

Tumor hypoxia is associated with relative radioresistance and aggressive biological behavior. Canine comparative oncology research has contributed meaningfully to the advancement of radiobiology research through tumor oxygenation studies. To validate canine cancer as a translationally relevant model of tumor hypoxia, Cline et al. detected the *in vivo* binding of a 2-nitroimidazole hypoxia (CCI-103F) marker in histochemical sections of canine tumors (40). The binding pattern was consistent with the expected location of hypoxic cells in tissues for which oxygen concentration gradients have been established by diffusion. The hypoxic fractions appeared in regions adjacent to necrosis, but also in regions free of necrosis. In addition to interest in hypoxic cells, populations of both non-cycling quiescent cells and rapidly-cycling proliferating cells can also influence tumor radioresponses. Zeman et al. investigated the relationships between hypoxia and proliferative status semi-quantitatively via immunohistochemical analysis of CCI-103F and proliferating cell nuclear antigen (PCNA), respectively, in canine tumor samples (41). Tumors with both high and low hypoxic and proliferative area fractions were identified; the hypoxic and proliferative cell populations overlapped to varying extents.

Direct, real-time quantification of tissue oxygenation was enabled by emergence of the Eppendorf method of direct oxygen partial pressure measurements. This technique, which involves intratumoral placement of polargraphic oxygen needle electrodes, opened the door for comparative veterinary trials characterizing the tumor microenvironmental effects of hypoxia in spontaneous canine tumors; it also allowed trials designed to investigate the impact of tumor oxygenation on treatment outcomes. Achermann et al. evaluated the oxygenation of canine soft tissue sarcomas via the Eppendorf method and determined that 44% of tumors had oxygenation measurements consistent with hypoxia (42). Soon after, trials were performed in dogs undergoing fractionated RT. Polarographic needle electrodes and OxyLite fluorescence probes were used to document the presence and changes of hypoxia during fractionated RT; 58% of the dog tumors in one study were hypoxic prior to treatment (43). The pO_2_ of initially hypoxic tumors remained unchanged during fractionated RT, whereas the pO_2_ decreased in initially normoxic tumors. Brurberg et al. evaluated pO_2_ fluctuations in spontaneous canine tumors prior to and during RT (44). It was found that overall oxygenation status differed substantially among the tumors, and RT had no consistent effect on overall oxygenation status. Fluctuations in pO_2_ were detected in both unirradiated and irradiated tumors, and those fluctuations were independent of the baseline tumor oxygenation status. This study was important as it demonstrated for the first time in canine cancer the dynamic changes in tumor oxygenation in spontaneous tumors over an extended time period. The influence of tumor oxygenation status on the response to RT was first described for spontaneous canine tumors by Bley et al. (45). Pretreatment oxygen level measurements in spontaneous canine tumors were correlated with local tumor response after RT; after curative-intent full-course irradiation, hypoxic tumors had a significantly shorter median progression-free interval and a shorter overall survival time compared to better oxygenated tumors.

Comparative canine oncology trials were instrumental to understanding how hyperthermia can be combined with RT to improve tumor control. A number of positive randomized studies in dogs provided initial evidence supporting the therapeutic benefit of such combinatorial therapy (46–48). In canine soft tissue sarcomas (STS), Vujaskovic et al. identified changes in tumor oxygenation, extracellular pH, and blood flow after hyperthermia (49). They also found that hyperthermia has biphasic effects on tumor physiologic parameters: lower temperatures tend to favor improved perfusion and oxygenation, whereas higher temperatures are more likely to cause vascular damage, leading to greater hypoxia.

## Emerging Uses of Dogs in Translational Radiation Research

### Imaging/Theranostics

**Figure 1:**
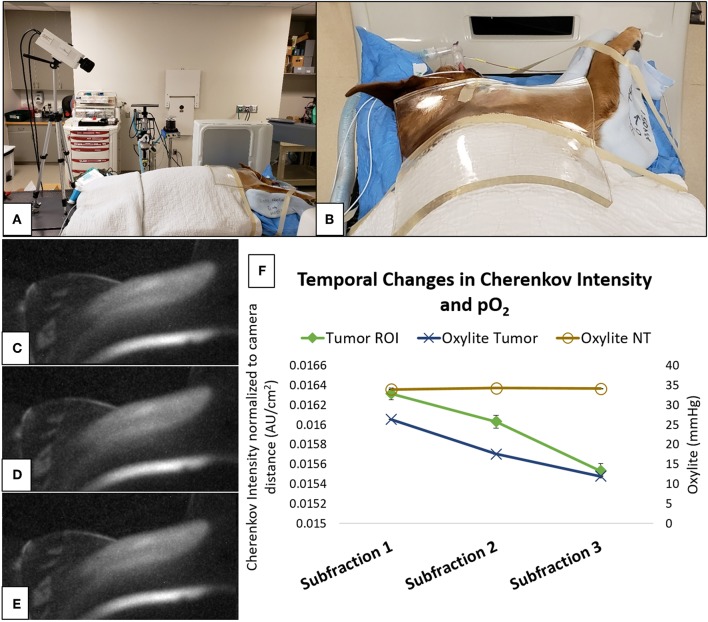
Cherenkov imaging represents a non-invasive method for quantification of tumor oxygenation during radiation delivery, and is currently being validated in a canine clinical trial (50). **(A)** camera setup for Cherenkov image acquisition during irradiation of a soft tissue sarcoma on the right shoulder of a dog, including a clear/colorless 1 centimeter thick bolus material overlying the tumor and used for radiation dose-buildup; **(B)** “camera's-eye” view of the irradiation target—fur overlying the tumor has been removed; **(C–E)** representative Cherenkov images taken during a single radiation fraction that was delivered using the setup depicted in **(A,B)**; **(F)** relative to stable normal tissue (NT) oxygenation, reductions in both Oxylite measurements and Cherenkov intensity within the tumor region of interest (ROI) demonstrate that the tumor became progressively more hypoxic during delivery of this 6 Gy radiation fraction. Subtle visible changes within the tumor ROI (the centrally-located light areas on Cherenkov images **C–E**) correspond to reductions in signal intensity (subfractions 1–3, respectively, on the graph) that were measured using digital image processing tools (images courtesy of Ashlyn Rickard, Duke University).

Canine comparative oncology studies that incorporate functional imaging technologies have been used to characterize the tumor microenvironment, improve target delineation, optimize biological dose delivery, and correlate imaging characteristics with clinical outcomes. Building upon the early oxygenation and radioresponse research which relied on tissue sampling or direct insertion of electrodes for measurements, functional imaging studies provide opportunities for serial, non-invasive, quantitative or semi-quantitative analyses of the tumor microenvironment without tissue disruption (Figure 1).

Various positron emission tomography (PET) radiotracers have been used in comparative oncology studies to characterize the tumor microenvironment. The glucose analog 2-deoxy-2-[^18^F]-Fluoro-D-glucose (FDG), a marker of glucose uptake, is the most commonly used PET tracer in clinical oncology, and canine cancer patients were among the earliest to be imaged with FDG PET (51). FDG PET/CT has become increasingly available in veterinary medicine (52), and it has been used to characterize tumor biology and treatment responses (53–57).

**Figure 2:**
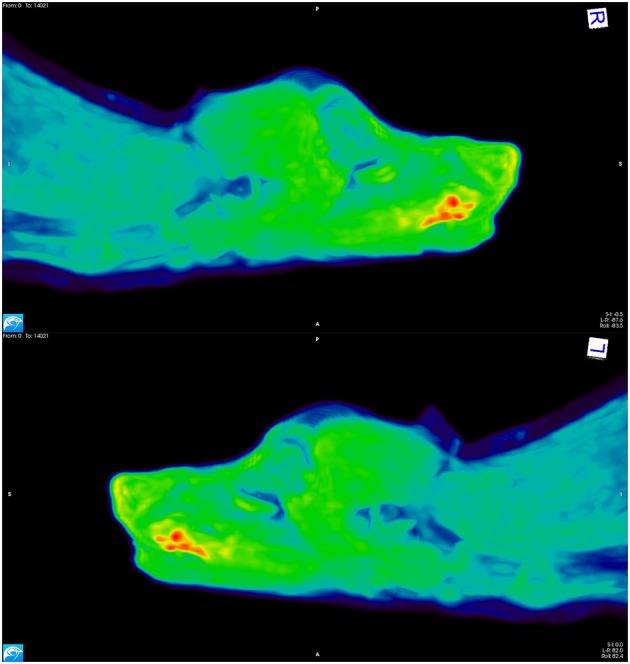
[^18^F]-FAZA PET scan images from a dog with a squamous cell carcinoma on the ventral aspect of the tongue. The areas depicted in red have high uptake of the tracer, indicating hypoxia; the yellow and green regions have intermediate uptake, and the blue areas have low tracer uptake, thus indicating the presence of well-oxygenated tissue.

As tumor hypoxia is associated with both radioresistance and tumor aggressiveness, PET-based approaches have been developed for measuring tumor hypoxia (Figure 2). Bruehlmeier et al. were the first to examine tumor hypoxia in canine STS using [^18^F]-fluoromisonidazole ([^18^F]-FMISO); FMISO tumor oxygenation measurements correlated well with Eppendorf electrode measurements (58). However, when evaluating tumor hypoxia via [^18^F]-FMISO in cats with fibrosarcomas, the polarographic pO_2_ measurements did not confirm PET results; this lack of concordance was attributed to extensive tumor necrosis, and heterogeneous patterns of hypoxia (59). An alternative hypoxia imaging tracer is ^64^Cu-ATSM. Hansen et al. performed a study to compare uptake characteristics of pimonidazole immunohistochemistry (IHC) to ^64^Cu-ATSM autoradiography, and to PET uptake levels of ^64^Cu-ATSM and [^18^F]-FDG in spontaneous canine sarcomas and carcinomas (60). Tumors with high levels of pimonidazole staining displayed high uptake of [^18^F]-FDG and ^64^Cu-ATSM; the regional distribution of ^64^Cu-ATSM and pimonidazole correlated with each other in heterogeneous tumor regions. The potential of using ^64^Cu-ATSM to characterize the tumor microenvironment longitudinally over time was evaluated in canine tumors by measuring tumor uptake and distribution characteristics between consecutive PET scans (61). In this study, ^64^Cu-ATSM uptake was also compared to uptake and spatial distributions of [^18^F]-FDG and dynamic contrast enhanced perfusion CT perfusion maps. ^64^Cu-ATSM uptake was positively correlated to FDG, signal was relatively stable between PET scans, and temporal changes were observed in hypo-perfused regions. ^64^Cu-ATSM PET/CT scan has also been used to detect hypoxia in feline head and neck squamous cell carcinoma (HNSCC), with PET/CT results verified by pimonidazole IHC and O_2_ detection probes (62).

Comparative canine oncology trials have supported investigations into the kinetics of established and novel PET/CT approaches, as well as characterization of the spatial distribution of these biological markers (63–65). Bradshaw et al. concurrently evaluated the predictive value of numerous quantitative imaging biomarkers derived from multitracer PET imaging in tumors before and during RT in dogs with sinonasal tumors (66). The strongest predictors of poor outcome were derived from fluorothymidine (FLT) imaging, a marker of proliferation. The combination of high mid-treatment standardized uptake values (SUV_max_) and large decreases in FLT signal from pretreatment to mid-treatment was associated with worse clinical outcome. In this study, neither FDG PET nor Cu-ATSM PET were predictive of outcome. PET/CT has also been utilized in comparative oncology studies to investigate the potential for radiation “dose painting,” which aims to improve therapeutic outcomes by increasing radiation dose in tumor regions that are identified as being at risk of relative radioresistance based upon imaging features (67, 68). Early results have been mixed.

**Figure 3:**
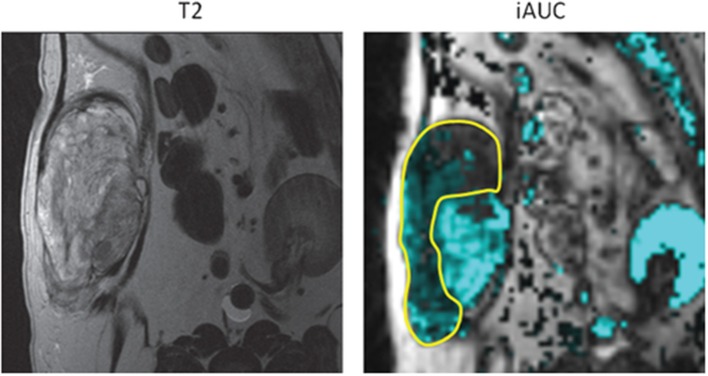
Canine soft tissue sarcoma. Pre-treatment quantification of iAUGC_100_ (initial area under the gadolinium concentration vs. time curve; this is a semi-quantitative parameter defined as a measure of the amount of contrast agent delivered to and retained by a tumor in a given time period). Outlined area represents the region with reduced perfusion and suspected hypoxic tissue. Radiation dose could be intensified to this area with the attempt to improve treatment outcome to suspected resistant tumor cell population. With permission from Boss et al. (69).

Dynamic contrast-enhanced magnetic resonance imaging (DCE-MRI) can be used to assess tumor physiology by exploiting abnormal tumor microvasculature (Figure 3). This enables quantitative assessment of tissue vessel density, integrity, and permeability (69). In a canine STS study, DCE-MRI was performed before and following the first hyperthermia treatment, and parameters associated with increased tumor perfusion were predictive for overall and metastasis-free survival (70). This was the first time that DCE-MRI was shown to be predictive of clinical outcome for STS. A subsequent study performed an integrative analysis of gene expression and diffusion weighted imaging (DWI) parameters, pre- and post-treatment, in dogs with STS treated with thermoradiotherapy. DWI is an MRI-based technique which quantifies the diffusion of water to characterize tumor tissue (71). Significant correlations were identified between gene expression and DWI. An unsupervised analysis of the gene sets revealed two clusters: (1) tumors with an increase in tissue water content (corresponding to an increased ADC on the DWI) after treatment showed increases in genes associated with tissue remodeling (e.g., IL1β, IL6, IL8, IL10) and inflammation (e.g., MMP1, TGFβ); (2) tumors with less change in the ADC had more signs of more mature vasculature (i.e., higher gene expression of CD31 and vWF). These observations demonstrated how one can link changes in tumor physiology to changes in gene expression. This work demonstrates how early changes in functional imaging parameters might be used to aid in prognostication. Furthermore, the authors provided a blueprint for how such data can be manipulated to identify potential new drug targets.

### DNA Damage Responses

The DNA damage response (DDR) is a complex network of pathways that responds to both endogenous and exogenous DNA damage. DDR deficiencies can be targeted for cancer therapy. For example, the DDR can be inhibited as a radiosensitization strategy. Additionally, because DNA damage drives chronic inflammation and various molecular and cellular pathways of the DDR activate immune signaling (72) and since checkpoint inhibitors may work best in the setting of tumors with high mutation load, DDR inhibition during RT can generate and preserve treatment-induced DNA damage which sensitizes the tumor to subsequent checkpoint blockade (73). There are many DDR components that can be inhibited to sensitize cancer cells to DNA damaging agents, including PARP, DNA-PK_cs_, ATM, ATR, Chk1, and Chk2.

Many novel agents that show great promise in mice ultimately fail to prove efficacious in humans. Despite improved rodent models, methodologies, and study designs, many factors still contribute to these failures (74). With specific regard to DDR modifiers, a recent review suggested that better predictive biomarkers are needed to identify patients that would benefit from treatment, and that better therapeutic response biomarkers are also needed to quantify the pharmacodynamic impact and clinical gains that are achieved in humans (75). Successful and efficient translation of DDR inhibitors will rely upon preclinical studies that are designed to evaluate appropriate endpoints that can also be measured in human trials; when possible, they should also determine whether synthetic lethality contributes to efficacy of the drug as a chemo-radiosensitizer.

Dogs have previously been proposed as a model for studying the DDR (76). Indeed, the intrinsic radiosensitivity of various canine tumors and tumors cell lines is increasingly well-understood (77, 78), and methods for measuring the canine DDR have also been developed, including validation of the comet assay and immunohistochemistry for phosphorylated H2AX (79).

Canine extremity osteosarcoma (OS) may be particularly useful in the development of novel DDR inhibitors that can be combined with RT—ATR, ATM, and DNA-PK inhibitors in particular. As with pediatric OS, tumor resection plus chemotherapy is the standard treatment for canine OS. However, because RT can also provide significant analgesia and local tumor control, and because tumor necrosis is a validated surrogate for local control of canine OS, an intriguing study design would be to treat OS-bearing dogs with chemoRT, with or without a novel DDR inhibitor, and subsequently pursue tumor removal to provide standard-of-care treatment to the dog, and to provide investigators access to the resected tumor, which would thus allow robust measurement of treatment effects. Furthermore, approximately half of canine OS tumors have aberrant p53 function, making it possible to compare the effects of chemo-radiosensitizing effects of ATR or DNA-PK inhibition in normal tissues and tumors that may or may not benefit from synthetic lethality (80, 81). Thus, by studying canine OS, investigators would be able to learn about both mechanisms of interaction and clinically relevant biomarkers that directly translate to a variety of human cancers in a manner that is essentially agnostic of tumor histology.

### Immuno-Radiotherapy

Advanced metastatic disease is the most common cause of death in human patients and is a significant cause of death in dogs as well (82). Cancer immunotherapy is not a new concept, but interest re-emerged when in 2010 a phase 3 clinical trial in people with metastatic melanoma showed a survival advantage for those treated with ipilimumab, a monoclonal antibody that targets CTLA-4 (83). Since then, the use of immunotherapy in human clinical trials has expanded rapidly, with some amazing successes. The problem remains that the majority of patients do not respond favorably; furthermore, unacceptable and sometimes fatal toxicities have occurred (84, 85).

The idea that RT could induce a tumor response distant from the irradiated field has been around since the 1950s when the so-called “abscopal effect” was first proposed (86). In the context of RT, the abscopal effect refers to regression of metastatic lesions that are distant from the primary tumor and irradiated field. These are rare events; one study found 46 cases reported in the literature between 1969 and 2014 (87). That the abscopal effect occurs secondary to an immune response was first demonstrated in a 2004 mouse study which characterized radiation-induced abscopal effects as being a T-cell dependent event (88). Since that time it has become obvious that the mechanism is actually more complex, and involves a multi-faceted immune response (89).

While local irradiation can result in immunogenic cell kill by releasing neoantigens and creating local inflammation, irradiation can also attract immunosuppressive cells into the tumor microenvironment. This includes myeloid-derived suppressor cells, M2 tumor-associated macrophages and T regulatory cells; this cellular response is associated with release of cytokines (e.g., TGF-β and IL-10) which causes local immunosuppression (90). Given the clinical rarity of measurably and clinically beneficial RT-induced immune responses, and because of the potential for post-irradiation immunosuppression, it is logical that patients may benefit from a combination of RT plus immunotherapeutics that can more reliably induce beneficial systemic immune responses (91).

The dog immune system has been fairly well-characterized and shows great homology to humans (92). Because of these similarities, pet dogs with cancer can provide a useful model when looking to translate potential new radioimmunotherapies from mouse studies to human clinical trials.

**Figure 4:**
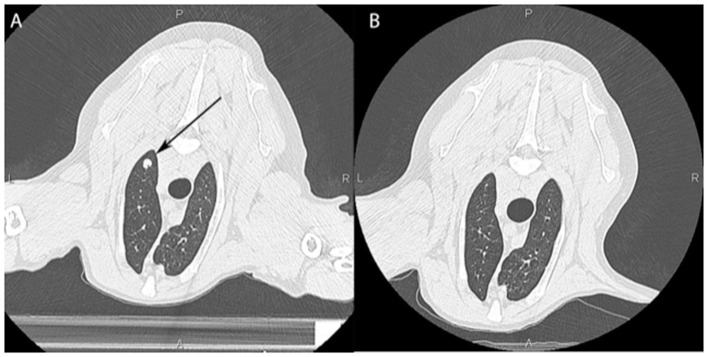
Computed tomographic image of a dog's lungs showing a metastatic lesion (arrow) taken prior to local irradiation of an oral tumor **(A)** and 3 months post treatment **(B)** showing an abscopal effect with near complete resolution of the mass.

Several small clinical trials using radio-immunotherapy protocols in dogs have been published. One recent example involved testing a novel immunotherapy combination in dogs with metastatic melanomas and sarcomas. Immediately after each fraction of primary tumor irradiation, intratumoral injections of canine CpG oligodeoxynucleotides [CpG ODNs; immune stimulatory toll-like receptor 9 (TLR9) agonists] were done; dogs were also orally dosed with 1-methyl-tryptophan [an indolamine-2,3 dioxygenase (IDO) inhibitor] (93). The idea was that localized tumor irradiation would induce immunogenic cell death, the CpG ODNs would stimulate an immune response, and the IDO checkpoint inhibitor would counter tumor-induced immunosuppression. The dog trial was paired with mouse studies which revealed a rebound immunosuppression after mice were treated with radiation or CpG ODNs alone. While all dogs showed a local response to irradiation, there were also abscopal responses in their metastatic lesions with one dog having a complete response, two having partial responses, one with stable disease and one with progressive disease (Figure 4). There was no toxicity beyond what was expected for a palliative course of localized RT. Interestingly, both circulating and tumoral T regulatory cells were decreased in the dogs who responded and increased in the dog with progressive disease—suggesting this as a potential biomarker. Due to the promising preliminary results and the lack of toxicity in dogs a clinical trial using this same strategy has now opened for people with advanced metastatic cancers (94).

In another study using an adoptive immunotherapy approach combined with RT, dog natural killer cells were isolated from peripheral blood (95). Those cells could be expanded and activated, and NK cells were capable of killing osteosarcoma tumor cells *in vitro*. Furthermore, cytotoxicity was improved when tumor cells were pretreated with ionizing radiation. This was repeated *in vivo* using canine patient-derived xenograft (PDX) tumors; adoptively-transferred canine NK cells delayed the growth of tumors in mice, and focal irradiation increased NK cell homing to these sarcoma xenografts. As part of the same study, a proof of concept clinical trial was carried out in 10 dogs with osteosarcoma. Treatment consisted of a course of palliative-intent RT followed by two intra-lesional injections of autologous activated canine NK cells. The NK cells were isolated, expanded, and activated *ex vivo* and supplemented with rhIL-2. The study demonstrated that NK cells persisted at the local tumor site for at least 1 week after injection. There was acceptable toxicity and in one of the cases there was resolution of a suspected metastatic lung nodule suggesting a possible abscopal effect.

Despite these early successes, there are several challenges in carrying out immune-RT trials in dogs, including: (1) limited availability of validated reagents, canine-specific monoclonal antibodies, and canine interleukins; and (2) limited access to properly staffed and equipped veterinary radiation oncology and RT centers (92). Work is ongoing to better characterize the canine immune system, and while many canine-specific antibodies are still not commercially available, this is improving. The majority of available checkpoint inhibitors are humanized monoclonal antibodies for which commercially available caninized versions do not exist. Furthermore, while it is possible to use recombinant human interleukins in dogs (they have been shown to be effective), there is concern that with prolonged use dogs could develop neutralizing antibodies and/or adverse immune reactions to the human-derived products. Thus, it will be difficult to fully realize the potential of this model until more immunotherapies become available for safe use in dogs.

## Future Directions

The existing experimental animal models (e.g., rodents, zebrafish) are, and will remain, vitally important for *in vivo* radiation oncology research. Yet, there are opportunities in using pet dogs that have not been fully explored; pet dogs with spontaneously arising tumors can better inform the use of RT for clinical management of various human cancers. All of the topics discussed above will continue to spawn new research. Some of the additional areas where companion animal studies could make an impact in human medicine include radiobiology studies looking at different dosing and fractionation schemes, *in vivo* dosimetry, and testing of newly developed radiation sensitizing and radiation mitigating agents.

Small animal irradiators used in rodent research are increasingly sophisticated, but remain unable to recapitulate medical linear accelerators in terms of beam energy and dose rate (96, 97). Because companion animal oncology patients are routinely treated using standard clinical linear accelerators, they are also readily available for studies that seek to better understand the radiobiological effects of such factors as spatial fractionation (e.g., GRID and Lattice RT) and dose rate (e.g., FLASH-RT) (98, 99). For example, canine tumors are particularly well-suited to understanding the underlying biology of FLASH-RT. This is because the apparent selective normal tissue sparing (vs. tumor sparing) achieved by FLASH-RT is likely dependent upon pO_2_; and as discussed above, because the size and growth rate of canine tumors is more similar to human cancers than experimentally-induced tumors in rodents, the oxygen (hypoxia) profiles of canine tumors also tend to be similar to those of human tumors. FLASH-RT also provides a particularly good example of how studies in companion animal species can be used as an intermediate step in scaling technologies from geometries that work for rodents, to those that would work well in the context of human tumors (100). In much the same way that treatment of canine intranasal tumors was used in the early development of helical Tomotherapy, these types of companion animal studies provide an excellent vehicle for testing the safety of, and establishing feasible clinical workflows for, novel treatment devices and approaches (101).

One area where dog studies may be particularly useful is understanding the interplay between radiation dose and immune responses. Some *in vivo* work suggests that the ideal fractional dose for radioimmunotherapy may be 6–8 Gy per fraction, rather than the more extreme doses that are commonly used in modern SRS/SBRT (89, 102). Interestingly, a recent study showed an inverse relationship between radiation dose and survival in humans having undergone RT for stage III non-small cell lung cancer; worsening outcomes with increasing radiation dose was counterintuitive but may have been attributable to delivery of higher radiation doses to tumor-infiltrating immune cells. Hypofractionation is commonly used in veterinary medicine both for palliation, and in SRS/SBRT; this practice pattern makes it relatively straightforward to interpose scientific research into clinical practice in order to use dogs as a model for studying the biology of hypofractionation.

Research in the field of therapeutic radiation physics could also benefit from a canine comparative radiation oncology approach. Because of similarities between human and canine anatomy and treatment approach, dogs can be a valuable model for validation of new techniques and methods; this use of canine comparative radiation oncology research has been exemplified for *in vivo* dosimetry (103).

There are also several limitations of the canine “model” that must be addressed. First, access to clinical outcomes data, and canine cancer tissues is limited; at the present time, there are no canine cancer registries in the United States, there are few well-validated canine cancer cell lines, and there are very few well-curated canine cancer tissue banks exist. Second, while dogs and their cancers more closely recapitulate the geometry of human cancers than do tumors of rodents, it should be noted that many pet dogs weigh far less than half the average human adult. Third, while there are many similarities between various human and canine malignancies, important differences also exist. For example, while human prostate cancer most often arises from the glandular acini, carcinomas of the canine prostate seem to most often be of urothelial origin. And although IDH1 and IDH2 mutations are frequent in human gliomas, they are not a consistent or common feature of the canine condition (104). The value of any model system is maximized by understanding its strengths and limitations; thus, moving forward, it will be of utmost importance to focus significant energy on describing the biology of canine cancers, and making rigorous comparisons with the analogous human conditions.

Though the field of canine comparative radiation oncology research is still in its infancy, canine studies have already helped advance the understanding of human tumor biology and treatment. Through continued efforts to improve our understanding of canine tumor biology, and careful application of the canine comparative oncology model, we expect that man's best friend will be key to reducing the global burden of cancer and improving cancer care.

## Author Contributions

MN, MK, and M-KB each contributed to the drafting and editing of this manuscript, and each also approved the final version.

### Conflict of Interest

The authors declare that the research was conducted in the absence of any commercial or financial relationships that could be construed as a potential conflict of interest.
